# Emulation of a Target Trial to Evaluate the Causal Effect of Palliative Care Consultation on the Survival Time of Patients with Hepatocellular Carcinoma

**DOI:** 10.3390/cancers13050992

**Published:** 2021-02-27

**Authors:** Tassaya Buranupakorn, Phaviga Thangsuk, Jayanton Patumanond, Phichayut Phinyo

**Affiliations:** 1 Department of Family Medicine, Chiang Rai Prachanukroh Hospital, Chiang Rai 57000, Thailand; tassayita@gmail.com (T.B.); Phaviga@gmail.com (P.T.); 2 Center for Clinical Epidemiology and Clinical Statistics, Faculty of Medicine, Chiang Mai University, Chiang Mai 50200, Thailand; jpatumanond@gmail.com; 3 Department of Family Medicine, Faculty of Medicine, Chiang Mai University, Chiang Mai 50200, Thailand; 4 Musculoskeletal Science and Translational Research (MSTR) Cluster, Chiang Mai University, Chiang Mai 50200, Thailand

**Keywords:** hepatocellular carcinoma, palliative care, supportive care, mean survival time

## Abstract

**Simple Summary:**

Hepatocellular carcinoma (HCC) is a common and aggressive liver cancer. As most patients are diagnosed during an incurable stage of the disease, they usually face great suffering during the end-of-life period. Palliative care can improve the patient’s quality of life and alleviate both physical and psychological symptoms. However, the discipline is underutilized due to a common misconception that it will accelerate the patient’s death. We emulated a hypothetical target trial to evaluate the causal effect of palliative care consultation on the survival time of patients diagnosed with HCC from retrospective observational data of a Thai tertiary care center. Although no clear survival benefit or harm was identified, palliative care consultation significantly reduced the use of unnecessary life-sustaining intervention, healthcare costs, and the risk of dying in the hospital among patients with HCC during their end-of-life period.

**Abstract:**

Palliative care has the potential to improve the quality of life of patients with incurable diseases or cancer, such as hepatocellular carcinoma (HCC). A common misconception of palliative care with respect to the patient’s survival remains a significant barrier to the discipline. This study aimed to provide causal evidence for the effect of palliative care consultation on the survival time after diagnosis among HCC patients. An emulation of a target trial was conducted on a retrospective cohort of HCC patients from January 2017 to August 2019. The primary endpoint was the restricted mean survival time (RMST) at 12 months after HCC diagnosis. We used the clone–censor–weight approach to account for potential immortal time bias. In this study, 86 patients with palliative care consultation and 71 patients without palliative care consultation were included. The adjusted RMST difference was −29.7 (95% confidence interval (CI): −81.7, 22.3; *p*-value = 0.263) days in favor of no palliative care consultation. However, palliative care consultation was associated with an increase in the prescription of symptom control medications, as well as a reduction in life-sustaining interventions and healthcare costs. Our findings suggest that palliative care consultation was associated with neither additional survival benefit nor harm in HCC patients. The misconception that it significantly accelerates the dying process should be disregarded.

## 1. Introduction

Hepatocellular carcinoma (HCC) is the most common primary cancer of the liver and a leading cause of cancer-related mortality worldwide [1]. In Thailand, HCC is highly prevalent, especially in the northern and the northeastern parts of the country, both endemic areas of viral hepatitis [2]. The overall prognosis is poor, with a reported survival probability of only 7–9% at 5 years in the Asian population [3]. A large proportion of HCC patients are diagnosed in their advanced stage with no available treatment option left [4], and they have to suffer from multiple debilitating symptoms from HCC itself and their underlying liver condition [5,6], which severely impairs their quality of life [7]. Integration of multidisciplinary palliative care into standard oncologic care can improve quality of life, alleviate physical symptoms, reduce psychological depression, reduce healthcare expenditure, or even prolong the survival of HCC patients, as already proven in other types of cancer [8,9,10,11]. Even though the benefits gained from palliative care referral seem attractive and are supported by standard practice guidelines [12,13,14], it is still underutilized in HCC patients [3,15].

One of the most significant barriers to implementing palliative care in practice is the stigmatization of the discipline by both the physicians and the patients [16,17]. Some patients and families might perceive that palliative care is only suitable for patients within the end-of-life stage or for cancer patients when curative therapy is no longer indicated [3,18]. Several recent studies and surveys revealed that a majority of gastroenterologists also held on to this misconception that palliative care should only be initiated when there was no role of active therapy in patients with end-stage liver disease [17,19]. Some physicians believe that palliative sedation therapy is the same as slow euthanasia or terminal sedation, which hasten death [20]. There was an increase in evidence that negates these misbeliefs over the past years [21,22,23,24]; however, most studies were conducted in patients with end-stage liver disease or other cancers. This study aimed to provide causal evidence for the effect of palliative care consultation on the survival time after diagnosis and healthcare utilization in patients with HCC through emulation of a target trial from observational data.

## 2. Materials and Methods

### 2.1. Study Design and Setting

Therapeutic research examining the effect of palliative care consultation on the survival of patients with HCC was conducted using data collected from electronic medical records of Chiang Rai Prachanukroh Hospital. The hospital is a tertiary care center with approximately 800 in-hospital beds and is located in the north of Thailand. In this study, we employed two methodologic approaches to answer our causal questions. For the first approach, approach A, we performed a conventional analysis of the retrospective observational cohort of patients with HCC by comparing the overall survival time after HCC diagnosis between patients who were consulted and not consulted for palliative care (i.e., ever consulted vs. never consulted). However, approach A is susceptible to immortal time bias. This type of bias usually arises when the point of treatment initiation (or the point of consultation) and the point to start of follow-up do not coincide. Thus, palliative consultation is observed only for patients who have survived for some time after HCC diagnosis, which artificially contributes to an inflated survival benefit of palliative care consultation. To prevent the occurrence of immortal time bias, we performed a second analytic approach, approach B, by emulating a hypothetical target trial from the original cohort of patients with HCC on the basis of the methods suggested by Maringe et al. [25]. Table 1 summarizes the hypothetical target trial protocol and the emulated cohort compared to the original cohort design. The Ethical Committee for Research in Human Subjects of Chiang Rai Hospital approved the study (CR 0032.102/EC013 CRH 085/62 In).

### 2.2. Data Collection

Data on patient demographic characteristics (age, gender, healthcare insurance), clinical characteristics (comorbidity), tumor characteristics (etiology of HCC, Barcelona Clinic Liver Cancer (BCLC) staging system for primary HCC, tumor size), Child–Turcotte–Pugh (CTP) score, previous HCC treatment (surgery, chemotherapy, radiotherapy), medications and intervention procedures used for symptom control or life-saving purposes during the last admission, and direct hospital costs during the last admission were collected from electronic medical records.

### 2.3. Eligibility Criteria

The study domain was adult patients diagnosed with hepatocellular carcinoma (HCC) at Chiang Rai Prachanukroh Hospital. The diagnosis of HCC was based on the principal diagnosis by the International Statistical Classification of Diseases and Related Health Problems 10 (ICD-10) code C22.0, malignant neoplasm of liver and hepatic bile ducts. All patient records and diagnoses were reviewed and verified by the investigators (P.P. and J.P.). Patients diagnosed with other cancers (e.g., cholangiocarcinoma) were excluded at this stage.

For approach A, all patients aged over 18 years old diagnosed at our hospital from January 2017 to August 2019 were included in the analysis. For approach B, the eligibility of the target trial was patients aged over 18 years old with a confirmed diagnosis of HCC at any BCLC staging and any level of performance status. No specific exclusion criteria were defined as the target trial was intended to be highly pragmatic.

### 2.4. Treatment Strategies and Assignment

In the original cohort, patients were classified into either the palliative consultation group (concurrent palliative and standard oncologic care) or the no palliative consultation group (only standard oncologic care) on the basis of documented consultation records or the ICD-10 code Z51.5, encounter for palliative care. Patients with HCC who did not have any documented palliative care consultation record during the study period were grouped in the standard oncologic care group.

Patients were categorized according to whether they were consulted or were not consulted for palliative care within 12 months of HCC diagnosis in the hypothetical target trial. We allowed a relatively long grace period of 12 months as our causal question was the following: Does palliative care consultation at any time within the first year after HCC diagnosis affect the patient’s survival? Thus, any consultation within the 12 months should be counted.

We cloned the included patients to imitate random allocation, which allowed us to assign each patient to both treatment strategies until their treatment strategy was confirmed. At randomization (ideally, at HCC diagnosis), we assumed that all patients had the same probability of being assigned to palliative care consultation or no palliative care consultation. Each patient entered both treatment strategies independent of their subsequent consultation status. To achieve this, we created two clones for each patient. We allocated one clone to each treatment strategy, which would double the sample size of the dataset and simultaneously balance all prognostic factors at baseline. In each treatment strategy, patient follow-up times were censored when they deviated from the planned protocol of treatment. For example, patients who were consulted for palliative care within 12 months were censored at their time of consultation in the no palliative care consultation (control) arm; patients who were not consulted for palliative care within 12 months were censored at 12 months in the palliative consultation arm.

### 2.5. Palliative Care and Advanced Care Plan

In our center, palliative care consultation is generally initiated by the attending physicians, either medical or surgical oncologists, to alleviate suffering symptoms or provide psychological support. After the consultation, palliative care team members, which comprise board-certified palliative care family physicians and nurses, would give an initial evaluation of the patient’s current status using methods recommended by the local Thai clinical practice guidelines for quality palliative care, which were adapted from those of the United States [26]. A Palliative Performance Scale (PPS) was obtained using the Adult PPS Suandok version [27], which was translated from the Victoria Hospice Society version. Symptom assessment was also performed using a Thai version of the Edmonton Symptom Assessment System (ESAS) [28]. Individualized treatment along with psychological support and pain control medications would be prescribed to patients who experienced disturbing symptoms, such as pain or dyspnea, following the World Health Organization essential medicines for palliative care [29]. Palliative care interventions are given concurrently with standard oncologic care with regular communications between the palliative care teams and the attending physicians to appropriately adjust the curative and palliative care intensity.

A meeting with the patients and their family members is held to set the goal of care. The team members would assist the patients and the families in making decisions regarding treatments during the end-of-life period. For patients who want to receive their end-of-life care at home, the team members would help prepare family members for essential patient care skills and provide them with the necessary equipment, such as a syringe driver and home oxygen concentrator, depending on the individual needs of the patients. Details regarding the advanced care plan of each patient would be documented and sent via Chiang Rai Smart Continuum of Care (COC) System, which is accessible to the local community hospitals within the catchment area, so that appropriate home care can be given and followed up by local healthcare providers.

### 2.6. Follow-Up and Endpoints

The patients were followed up from HCC diagnosis until death. The primary study endpoint was the survival time of the patients, starting from the date at diagnosis of HCC to the death date. The data on time and date of death were obtained and verified from the hospital discharge summary for patients who died in the hospital or from the Thailand Civil Registration Office for patients who died at home. Secondary endpoints were total direct costs of hospital admissions during the last hospital admission, administration of life-sustaining treatment in the last hospital admission, such as endotracheal intubation, cardiopulmonary resuscitation, and inotropes, prescription of symptom control medications during the last hospital admission, and place of death.

For approach A, the event contributed to the treatment strategy if the patients were initially classified in the original cohort (without cloning and censoring). This manner of patient classification often leads to immortal time bias in a time-to-event analysis. For approach B, the event contributed only to the treatment strategy in which the patient was still uncensored when the event occurred. This classification approach would correct for immortal time bias. Given a hypothetical example case of a female patient diagnosed with HCC who might be consulted if she could survive beyond 6 months after diagnosis but died 3 months after diagnosis, the death event of this woman would be attributed to only the no palliative care consultation strategy in approach A. On the other hand, the death event of this woman and her clone would contribute equally to both treatment strategies in approach B.

### 2.7. Statistical Analysis

All statistical analyses were performed using Stata 16 (StataCorp, College Station, TX, USA). Frequency and percentage were used to describe categorical data. Mean and standard deviation or median and interquartile range were used to describe numerical data, as appropriate.

#### 2.7.1. Inverse Probability Weighting

For approach A, we used inverse probability treatment weighting (IPTW), which is a type of propensity score method [30,31], to account for potential selection bias, confounding by indication, and confounding by contraindication [31]. Multivariable logistic regression modeling was used to predict the probability of being and not being in a palliative care consultation group for each specific patient. The following features were included in the logistic model: gender, age, type of health insurance, comorbidity, tumor etiology, BCLC staging, CTP score, tumor size, porto-venous involvement, and HCC specific treatment received. Other clinically relevant parameters at HCC diagnosis, such as serum albumin, serum bilirubin, prothrombin time, the presence of ascites, or hepatic encephalopathy, were not included in the model to avoid clinical and statistical collinearity with the CTP score [32]. These probabilities were then used for creating inverse probability weights to construct a weighted cohort of patients with similar baseline characteristics.

For approach B, as the decision for palliative care consultation might be based on several demographic or clinical characteristics usually associated with the outcome, the artificial censoring during follow-up introduced selection bias [33]. We addressed this serious issue by using inverse probability of censoring weighting (IPCW) [34,35], which weighted patients remaining in the risk set to maintain the comparability of two treatment strategies throughout the grace period and follow-up. We used a treatment-specific Cox’s proportional hazard regression to predict the probability of censoring mechanism for each treatment strategy. All potential prognostic factors and confounders were included in the Cox’s model according to clinical knowledge (i.e., gender, age, type of health insurance, comorbidity, tumor etiology, BCLC staging, CTP score, tumor size, porto-venous involvement, and HCC specific treatment received). Then, we derived the censoring weights by inversing the predicted probabilities, which was done separately for each treatment strategy.

Standardized difference (STD) was used to quantify the magnitude of differences in patient characteristics between the two groups after weighting. An absolute STD of more than 10% was considered a significant difference between groups [36]. Any variables that remain imbalanced after weighting would be adjusted for double robustness in the weighted analysis model [37,38].

#### 2.7.2. Primary Endpoint

For the primary study endpoint, the restricted mean survival time (RMST) difference was used to compare the mean survival times between the two groups [39]. To estimate the RMST for each group, we used a weighted flexible parametric survival regression to model the log cumulative hazards with three degrees of freedom for baseline hazard distribution and one degree of freedom for time–treatment interaction [40]. Three time points were chosen to calculate the RMST as follows: 90 days, 180 days, and 365 days. For approach A, the analysis would be considered as-treated, whereas for approach B the analysis would be regarded as per-protocol. To correctly estimate the variance for the treatment effect in approach B, a bootstrapping procedure with 500 replicates was used to derive a valid 95% confidence interval of the RMST difference.

A sensitivity analysis of the primary endpoint was performed by excluding patients who were consulted for palliative care during the terminal stage of their illness before initiating the cloning–censoring–weighting procedure, as the main objective for palliative care consultation during this period is not to prolong the patient’s survival but to alleviate and control end-of-life symptoms. We applied the following definitions to exclude the patients in each separate analysis: (1) patients who were consulted and did not survive 15 or 30 days after consultation; (2) patients who were consulted within 15 or 30 days of diagnosis. We hypothesized that the exclusion of these patients would not affect the overall estimates and that palliative consultation for patients with HCC at any stage of their disease does not affect the survival of the patients.

#### 2.7.3. Secondary Endpoints

For the secondary endpoints, we used a weighted generalized linear model with adjustment of baseline prognostic factors and confounders that showed a significant difference after inverse probability weighting to examine differences in the proportion of life-sustaining intervention done in the last admission, the proportion of symptom control medications prescribed, and place of death. Weighted median regression was used to model the difference in direct costs during the last hospital admission.

## 3. Results

A total of 157 patients diagnosed with HCC were included in the original cohort. The median follow-up time of the cohort was 44 (interquartile range: 14–169) days with no censored observation. There were 86 patients with documented palliative care consultation and 71 patients without palliative care consultation during the follow-up period. Table 2 compares the demographic, clinical, and tumor characteristics between patients who were consulted and not consulted for palliative care. Details on the subcomponents of the CTP score and alpha-fetoprotein (AFP) are described in Table S1 (Supplementary Materials). Almost all baseline demographic, clinical, and tumor characteristics were different between the two groups except for the presence of diabetes mellitus, hypertension, and chronic kidney disease as a comorbidity, and hepatitis B virus (HBV) infection as a tumor etiology (Table 2). The most significantly different characteristic between the two groups was the BCLC staging (STD +0.502). A higher proportion of terminal-stage patients was identified in the palliative care consultation group. Only 23 (14.7%) of the patients received at least one HCC specific treatment (15.1% in palliative care consultation group vs. 14.1% in no palliative care consultation; *p* = 1.000).

In the crude analysis, the median survival time was 28 (95% confidence interval (CI): 17, 63) days in the group with no palliative care consultation and 54 (95% CI: 42, 90) days in the group with palliative care consultation. The median time to the consultation was 28 (95% CI: 14, 49) days (Figure S1, Supplementary Materials). In the palliative care consultation group, 47 (54.7%) patients were consulted within the first 30 days of diagnosis of HCC, whereas only 12 (14.0%) were consulted after 1 year.

### 3.1. Primary Endpoint

**Figure 1:**
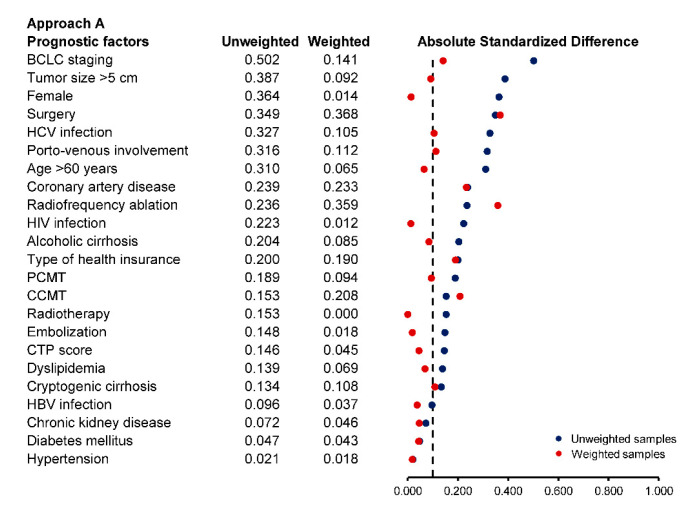
Balance in prognostic factors at baseline for approach A (conventional cohort analysis) based on absolute standardized difference in the unweighted and weighted samples.

In approach A, we used inverse probability weighting to eliminate potential selection bias at baseline. Most prognostic factors were well balanced after weighting except for the type of health insurance, coronary artery disease as a comorbidity, cryptogenic cirrhosis as an etiology of HCC, and surgical resection, conventional chemotherapy, radiotherapy, and radiofrequency ablation as HCC treatment (Figure 1). These remaining imbalanced factors were included in the weighted RMST regression model for double adjustment. The RMST at 365 days was 111.0 (95% CI: 92.8, 129.1) days in the palliative care consultation group and 105.9 (95% CI: 78.5, 133.3) days in no palliative care consultation group, with an adjusted RMST difference of +5.0 (95% CI: −23.8, 33.9; *p*-value = 0.732) days in favor of palliative care consultation. There was also no significant difference in the RMST at 90 days and 180 days (Table 3).

**Figure 2:**
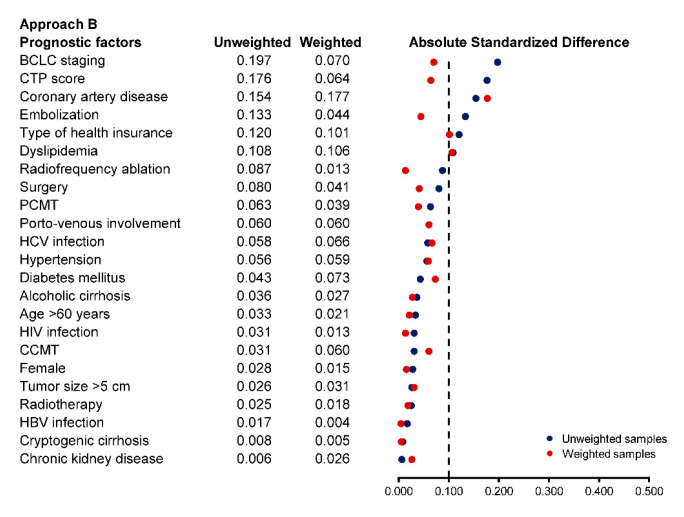
Balance in prognostic factors at 12 months after diagnosis for approach B (emulated cohort) based on absolute standardized difference in the unweighted and weighted clone samples.

In approach B, all 157 patients were categorized into palliative care consultation if consulted within 12 months and no palliative care consultation if they were not consulted within 12 months of HCC diagnosis. Then, they were cloned to eliminate the imbalance in baseline prognostic factors. After artificial censoring during the grace period, there was some baseline imbalance between groups at 12 months in BCLC staging, coronary artery disease, embolization, type of health insurance, and dyslipidemia. The derived censoring weights were able to balance the differences between the two groups except for the type of health insurance, coronary artery disease, and dyslipidemia (Figure 2). In the weighted analysis, the RMST at 365 days was 120.3 (95% CI: 96.2, 144.5) days in the palliative care consultation group and 150.0 (95% CI: 102.3, 197.7) days in no palliative care consultation group, with an adjusted RMST difference of −29.7 (95% CI: −81.7, 22.3; *p*-value = 0.263) days in favor of no palliative care consultation. There was no significant difference in the RMST at 90 days and 180 days (Table 3).

**Figure 3:**
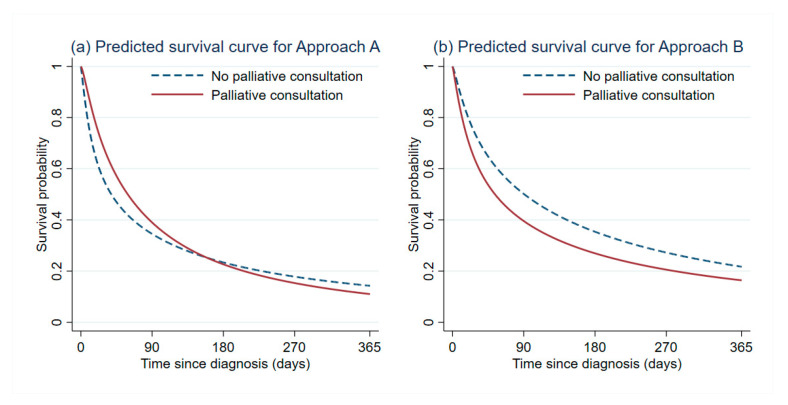
Predicted survival curve based on flexible parametric survival regression for approach A and approach B (limiting the analysis at 1 year after HCC diagnosis).

The predicted survival curves from both approach A and approach B are shown in Figure 3 to benchmark the results between a conventional analysis of the original patient cohort and a modern analysis of the emulated target trial. Table S2 (Supplementary Materials) presents the sensitivity analysis results to examine the robustness of our RMST difference estimates. No statistical significance in the RMST was identified between the two treatment strategies at every time point in all sensitivity analyses.

### 3.2. Secondary Endpoints

For the secondary endpoints, both analytic approaches yielded consistent significant results in favor of palliative care consultation (Table 4), except for the direct cost and prescription of medication for controlling dyspnea. During their last hospital admission, patients who were consulted for palliative care were less likely to receive life-sustaining interventions, including endotracheal intubation, cardiopulmonary resuscitation, and inotropic drugs or vasopressors. The prescription of symptom control medications was significantly higher in patients with palliative care consultation than patients without palliative care consultation. More than half of the patients consulted for palliative care chose to die at home, and only about 28 (32.6%) decided to die at their nearby community hospitals.

## 4. Discussion

This study found that palliative care consultation was associated with neither significant survival benefit nor harm among patients with HCC. Although the trend of RMST difference at 365 days was not in favor of palliative care consultation, a definite negative impact of palliative care on the patient’s survival could not be concluded due to the wide confidence interval of the estimate. However, significant benefits of palliative care consultation other than survival were identified, such as a reduction in the proportion of patients dying in hospitals, the direct hospital cost, and the administration of life-sustaining procedures within the last hospital admission of the patients. Palliative care consultation was also associated with an increase in the prescription of symptom control medications.

HCC is an aggressive tumor, ranked as the second cause of cancer-related mortality among the Thai population [2]. As most HCC patients are diagnosed at a later stage of the disease when curative treatments (i.e., surgical resection) are no longer indicated, the overall median survival time is typically less than 1 year [41,42]. In our data, which were obtained from a tertiary care hospital, only 14.7% were given HCC-specific treatment. Only 3% underwent surgical intervention, which was even lower than the figure reported in the nearby University hospital (7%) [43]. In this situation, where most patients have incurable disease with progressive symptoms, integration of palliative care with standard oncologic treatment is appealing. However, several barriers impede its use in clinical practice, such as the stigmatization of palliative care as a treatment for the hopeless with a potential to decrease survival [44,45,46]. Thus, a specific question of whether palliative care consultation affects the survival of HCC patients must be answered to provide convincing evidence to both the practitioners and the patients.

According to our primary results, the mean survival time was minimally shorter in the palliative care consultation group; however, no statistical significance was identified at every time point. The RMST differences were robust in the sensitivity analyses that excluded patients who were consulted soon after diagnosis or patients in their end-of-life period where survival benefits should not be expected [24]. Thus, no clear survival benefit or harm of consulting HCC patients for palliative care could be drawn. Even though our primary question could not be explicitly answered in terms of survival, other clinically relevant benefits were found. It is important to note that prolonging survival time should not be considered a primary goal of palliative care consultation; rather, its goals are to improve quality of life, alleviate suffering symptoms, and support patients and families through their difficult times [47]. This study confirmed that palliative care consultation in HCC patients leads to a significant reduction in life-sustaining intervention, healthcare resource utilization, and cost, which was already proven in other conditions [23,48,49,50,51].

Our findings suggest that palliative care consultation or referral did not improve survival in patients with HCC, unlike other cancers [24,47,52], which could be explained by the fact that HCC in its advanced stage is an incurable disease with significantly poor survival [5]. Most HCC patients also have underlying chronic liver disease or cirrhosis, which further contributes to the poor prognosis. It was reported that cirrhotic complications accounted for up to 40% of death in patients with unresectable HCC [6]. Another critical factor affecting the survival was the level of healthcare services accessible to the patients and the available infrastructure for delivering HCC-directed therapy (e.g., liver resection, radiotherapy, ablation, and embolization). In Thailand, most hospitals cannot provide all treatment modalities for HCC, including ours. For these reasons, expecting additional benefit from palliative care consultation in HCC patients might not be plausible. However, this must not be construed as a discouragement for patient referral and engagement to palliative care.

Our study emulated a hypothetical target trial that directly answers the causal question from the observational cohort of patients with HCC using the clone–censor–weight approach [25,53]. By realigning the point of eligibility and the point of treatment initiation (i.e., palliative care consultation) to be in the same place, we avoided a self-derived selection and immortal time bias [33]. We benchmarked the results of analytic approach A and approach B to show how these biases may affect our results. Approach A was seriously affected by immortal time bias as those ever-consulted groups had a guaranteed survival period while the other did not.

Our study carries several limitations that must be elaborated. First, the analysis was based on retrospective data with a small number of HCC patients in a single tertiary care center, limiting the generalizability of our results to other settings with different levels of care. Second, we did not include data on quality-of-life measurements and patient satisfaction within the analysis. These data were only available for patients who were consulted for palliative care services. However, this was not essential as our primary question did not concern these subjective aspects. Third, healthcare utilization and healthcare costs were only collected and summarized from the latest patient admission due to constraints on the data availability. Fourth, we defined palliative care consultation as a fixed treatment strategy (i.e., once started, always started) and did not conduct a separate analysis for inpatient and outpatient consultation. The compliance of the patient to the palliative care protocol was also not evaluated. Lastly, even after seemingly adequate statistical adjustment via inverse probability weighting in both analytic approaches, it is likely that the estimates might still have been affected by residual confounding due to unobserved variables. Further prospective studies or randomized trials are required to confirm our findings and examine the potential benefits in other aspects of palliative care consultation for HCC patients.

## 5. Conclusions

Our findings suggest that palliative care consultation was associated with neither additional survival benefit nor harm among patients with HCC but was associated with an increase in the prescription of symptom control medications to ease the dying process, which is the prime goal of palliative care during the end-of-life period. Palliative care consultation was also associated with a reduction in nonbeneficial life-sustaining interventions and healthcare costs. Palliative care consultation should be encouraged more in patients with HCC regardless of their current stage and performance status. The misconception of accelerating the dying process should be disregarded as survival benefit is not the primary goal of palliative care treatment.

## Figures and Tables

**Table 1:** Study protocol for target trial emulation comparing to the original patient cohort.

Component	Target Trial	Emulated Cohort	Original Cohort
Design	Randomized controlled, concurrent trial of patients with HCC.	Emulated target trial from observation cohort of patients with HCC.	Retrospective observational cohort of patients with HCC.
Aim	Estimate the effect of palliative care consultation within 12 months of HCC diagnosis on survival time of the patients.	Same as Target Trial.	Same as Target Trial.
Eligibility	Patients with HCC aged ≥18 years old diagnosed at any BCLC stage with any level of performance status.	Same as Target Trial.	Patients with HCC aged ≥18 years old who were diagnosed at CRH during January 2017 to August 2019.
Exclusions	Patients who died from non-cancer specific causes, such as traumatic accidents.	Same as Target Trial..	Same as Target Trial.
Treatment strategies	1. Palliative care consultation within 12 months of HCC diagnosis.	Same as Target Trial.	1. Palliative care consultation after HCC diagnosis.
	2. No palliative care consultation within 12 months of HCC diagnosis.		2. No palliative care consultation after HCC diagnosis.
Treatment assignment	Patients were randomly assigned to either strategy.	Trial emulation was performed via cloning of patients in both arms and assigning appropriate censor definition.	Patients were classified into either group on the basis of documented consultation records or ICD-10 code.
Treatment implementation	None.	12 months grace period.	Consulted or never consulted.
Outcome	All-cause mortality at 1 year after HCC diagnosis.	Same as Target Trial.	Same as Target Trial.
Type of outcome	Survival time.	Same as Target Trial.	Same as Target Trial.
Follow-up	Follow-up started at diagnosis, equivalent to treatment assignment and treatment initiation.	Follow-up started at diagnosis, which might not have corresponded with the initiation of palliative care consultation.	Follow-up started at diagnosis, which might not have corresponded with the initiation of palliative care consultation.
Censoring	Loss to follow-up, administrative censoring.	Same as Target Trial.	Same as Target Trial.
Adjustment variables	Age at diagnosis, gender, health insurances, comorbidity, tumor characteristics (etiology of HCC, BCLC staging, tumor size, porto-venous involvement), HCC specific treatment received.	Same as Target Trial.	Same as Target Trial.
Causal contrast	Per-protocol analysis.	Per-protocol analysis. Intended treatment could not be identified from the data. Trial emulation would censor patients, including clones, who deviated from their assigned protocol at the time of deviation.	As-treated analysis. Patients with HCC who did not have any documented record of palliative care consultation during the study period were grouped in the standard oncologic care group.
Statistical analysis (primary endpoint)	Differences in the restricted mean survival time among treatment arms at 90, 180, and 365 days after diagnosis.	Same as Target Trial.	Same as Target Trial.

Abbreviations: BCLC, Barcelona Clinic Liver Cancer staging; CRH, Chiang Rai Prachanukroh Hospital; HCC, hepatocellular carcinoma; ICD, International Statistical Classification of Diseases and Related Health Problems.

**Table 2:** Demographic, clinical, and tumor characteristics and HCC treatment of the original patient cohort.

Characteristics	Original Patient Cohort (*n* = 157)				
	Palliative Consultation (*n* = 86)		No Palliative Consultation (*n* = 71)		STD (%)
	n	(%)	n	(%)	
**Demographic characteristics**					
Female	20	(23.3)	7	(9.9)	−0.364
Age > 60 years	52	(60.5)	32	(45.1)	−0.310
Age (years, mean ± SD)	61.5	±12.1	59.5	±12.9	−0.159
Type of health insurance					
UC	38	(44.2)	31	(43.7)	−0.200
SSS	35	(40.7)	30	(42.3)	
CSMBS	9	(10.5)	9	(12.7)	
Self-paid	4	(4.7)	1	(1.4)	
**Clinical characteristics**					
Comorbidity					
Diabetes mellitus	11	(12.8)	8	(11.3)	−0.047
Hypertension	21	(24.4)	18	(25.4)	+0.021
Dyslipidemia	8	(9.3)	4	(5.6)	−0.139
Chronic kidney disease	5	(5.8)	3	(4.2)	−0.072
Coronary artery disease	0	(0)	2	(2.8)	+0.239
**Tumor characteristics**					
Etiology of HCC					
HBV infection	38	(44.2)	28	(39.4)	−0.096
HCV infection	17	(19.8)	6	(8.5)	−0.327
HIV infection	2	(2.3)	5	(7.0)	+0.223
Cryptogenic cirrhosis	3	(3.5)	1	(1.4)	−0.134
Alcoholic cirrhosis	20	(23.3)	23	(32.4)	+0.204
BCLC staging					
A (early stage)	3	(3.5)	2	(2.8)	+0.502
B (intermediate stage)	14	(16.3)	14	(19.7)	
C (advanced stage)	6	(7.0)	8	(11.3)	
D (terminal stage)	59	(68.6)	35	(49.3)	
Unknown	4	(4.7)	12	(16.9)	
Tumor size > 5 cm	63	(81.8)	37	(64.9)	−0.387
Porto-venous involvement					
Presence	46	(53.5)	27	(38.0)	+0.316
Unknown	10	(11.6)	12	(16.9)	
CTP score§					
A	12	(14.1)	11	(15.5)	+0.146
B	17	(20.0)	18	(25.4)	
C	56	(65.9)	42	(59.2)	
**HCC-specific treatment**					
CCMT	1	(1.2)	0	(0)	−0.153
PCMT	4	(4.7)	1	(1.4)	−0.189
Surgery	5	(5.8)	0	(0)	−0.349
Embolization	7	(8.1)	9	(12.7)	+0.148
Radiotherapy	1	(1.2)	0	(0)	−0.153
Radiofrequency ablation	5	(5.8)	1	(1.4)	−0.236

Abbreviations: BCLC, Barcelona Clinic Liver Cancer staging; CCMT, conventional chemotherapy; CSMBS, Civil Servant Medical Benefit Scheme; CTP, Child–Turcotte–Pugh; HBV, hepatitis B virus; HCV, hepatitis C virus; HIV, human immunodeficiency virus; NA, not applicable; PCMT, palliative chemotherapy; SD, standard deviation; STD, standardized difference; SSS, Social Security Scheme; UC, Universal Coverage. ^§^ Data on CTP score was only available for 156 patients.

**Table 3:** Primary study endpoints (restricted mean survival time) via two analytic approaches.

	Palliative Consultation		No Palliative Consultation				
	RMST		RMST		Adjusted RMST Difference		*p*-Value
	Mean (days)	95% CI	Mean (days)	95% CI	Mean (days)	95% CI	
**Approach A: Original Cohort**							
t = 90	55.3	49.5, 61.0	47.1	38.2, 56.0	8.2	−2.2, 18.5	0.121
t = 180	81.9	71.2, 92.6	72.5	56.1, 88.8	9.4	−9.6, 28.4	0.332
t = 365	111.0	92.8, 129.1	105.9	78.5, 133.3	5.0	−23.8, 33.9	0.732
**Approach B: Emulated Cohort**							
t = 90	52.5	47.1, 58.1	61.3	52.1, 70.5	−8.7	−19.0, 1.6	0.098
t = 180	81.8	67.0, 93.6	99.0	76.3, 121.8	−17.3	−41.5, 7.0	0.163
t = 365	120.3	96.2, 144.5	150.0	102.3, 197.7	−29.7	−81.7, 22.3	0.263

Abbreviations: CI, confidence interval; RMST, restricted mean survival time; No, number; t, time point for RMST analysis.

**Table 4:** Secondary study endpoints in the original cohort and the emulated cohort.

	Palliative Consultation (*n* = 86)		No Palliative Consultation (*n* = 71)		Multivariable Analysis						
					Original Cohort			*p*-Value	Emulated Cohort		*p*-Value
	*n*	(%)	*n*	(%)	Clinical Parameters	Adjusted Effect	95% CI		Adjusted Effect	95% CI	
**Last Hospital Admission**											
Direct costs (USD, median (IQR))	457	(205, 1253)	706	(414, 1279)	Median difference	−158.0	−504.4, 188.5	0.369	−317.5	−396.0, −239.0	<0.001
**Life Sustaining Intervention**											
ETT	3	(3.5)	40	(56.3)	Risk difference (%)	−0.45	−0.60, −0.31	<0.001	−0.50	−0.68, −0.31	<0.001
CPR	0	(0)	11	(15.5)		−0.09	−0.14, −0.3	0.001	−0.14	−0.26, −0.02	0.025
Inotropes	3	(3.5)	32	(45.1)		−0.36	−0.49, −0.23	<0.001	−0.46	−0.63, −0.28	<0.001
**Prescription of Symptom Control Medications**											
Dyspnea	41	(47.7)	17	(23.9)	Risk difference (%)	0.16	−0.01, 0.33	0.062	0.24	0.04, 0.44	0.017
Pain	75	(87.2)	15	(22.5)		0.58	0.42, 0.73	<0.001	0.66	0.51, 0.82	<0.001
Constipation	76	(88.4)	16	(22.5)		0.57	0.42, 0.73	<0.001	0.66	0.51, 0.82	<0.001
**Place of Death**											
Hospital	28	(32.6)	57	(80.3)		−0.41	−0.57, −0.24	<0.001	−0.49	−0.67, −0.31	<0.001

Abbreviations: CI, confidence interval; ETT, endotracheal intubation; CPR, cardiopulmonary resuscitation; CRH, Chiang Rai Prachanukroh hospital; SD, standard deviation; USD, United States Dollars.

## Data Availability

The datasets used and/or analyzed during the current study are available from the corresponding author on reasonable request.

## Supplementary Materials

The following are available online at https://www.mdpi.com/2072-6694/13/5/992/s1: Figure S1. Histogram visualizing the distribution of time to palliative care consultation (day) in the dataset; Table S1. Clinical and laboratory parameters at HCC diagnosis of the original patient cohort (*n* = 157); Table S2. Sensitivity analysis results based on the emulated cohort.
