# Supplementary material for: Emulation of a Target Trial to Evaluate the Causal Effect of Palliative Care Consultation on the Survival Time of Patients with Hepatocellular Carcinoma
Source: Cancers (Basel). 2021 Feb 27;13(5):992. doi: 10.3390/cancers13050992 (PMC7956840; doi:10.3390/cancers13050992)
Supplement: Supplementary file 1 [file cancers-13-00992-s001.pdf]

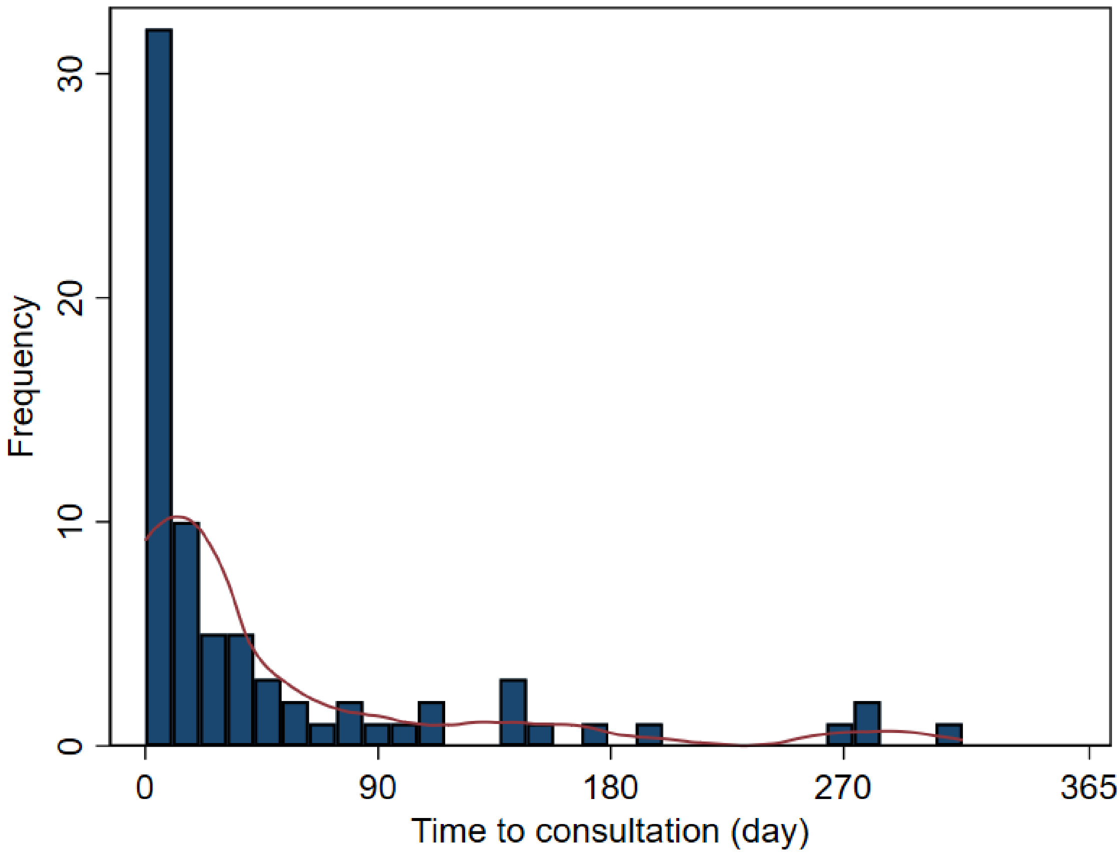

Figure S1. Histogram visualizing the distribution of time to palliative care consultation (day) in the dataset

Supplementary Table S1 Clinical and laboratory parameters at HCC diagnosis of the original patient cohort (n=157)

Characteristics	Missing data (n, %)	Original patient cohort (n=157)				
		Palliative consultation (n=86)		No palliative consultation (n=71)		STD (%)
		n	(%)	n	(%)	
CTP score	1 (0.6)					
A		12	(14.1)	11	(15.5)	+0.146
B		17	(20.0)	18	(25.4)	
C		56	(65.9)	42	(59.2)	
Albumin	1 (0.6)					
>3.5 g/dL		48	(56.5)	43	(60.6)	+0.181
2.8-3.5 g/dL		28	(32.9)	18	(25.4)	
<2.8 g/dL		9	(10.6)	10	(14.1)	
Bilirubin	1 (0.6)					
<2 mg/dL		26	(30.6)	32	(45.1)	+0.316
2-3 mg/dL		15	(17.6)	12	(16.9)	
>3 mg/dL		44	(51.8)	27	(38.0)	
Prothrombin level	23 (14.7)					
>6 seconds prolonged		46	(63.0)	22	(36.1)	+0.600
4-6 seconds prolonged		13	(17.8)	13	(21.3)	
<4 seconds prolonged		14	(19.2)	26	(42.6)	
Ascites§	3 (1.9)					
None		20	(23.8)	24	(34.3)	+0.285
Mild to moderate		30	(35.7)	26	(37.1)	
Severe		34	(40.5)	20	(28.6)	
Encephalopathy	1 (0.6)					
None		74	(87.1)	53	(74.6)	+0.436
Mild to moderate		4	(4.7)	13	(18.3)	
Severe		7	(8.2)	5	(7.0)	
AFP (ng/mL)	33 (21.0)					
mean±SD		14036	±55127	9967	±30403	-0.091
Median (IQR)		355	(24, 14366)	338	(11, 17051)	

Abbreviations: AFP, alpha-fetoprotein; CTP, Child-Turcotte-Pugh; STD, standardized difference.

Supplementary Table S2 Sensitivity analysis results based on emulated cohort.

	Adjusted RMST difference		P-value
	Mean (days)	(95%CI)	
Excluding patients who were consulted within 15 days after diagnosis			
t=90	-4.8	-14.4, 4.9	0.332
t=180	-14.0	-33.8, 5.8	0.166
t=365	-36.5	-79.7, 6.8	0.098
Excluding patients who died within 15 days after consultation			
t=90	-6.5	-15.1, 2.0	0.134
t=180	-17.8	-41.4, 5.8	0.139
t=365	-39.9	-94.7, 14.9	0.154
Excluding patients who were consulted within 30 days after diagnosis			
t=90	-3.3	-13.4, 6.7	0.514
t=180	-11.0	-31.0, 9.0	0.283
t=365	-31.2	-75.0, 12.7	0.163
Excluding patients who died within 30 days after consultation			
t=90	-3.8	-10.3, 2.7	0.251
t=180	-16.6	-38.3, 5.1	0.133
t=365	-48.9	-102.9, 5.2	0.076

Abbreviations: CI, confidence interval; RMST, No, number; Restricted mean survival time; t, time point for RMST analysis.
